# Repurposing of Chinese Medicine Extract against *Staphylococcus Aureus*: Assessing the Antibacterial and Anti-Transfer Activity of Plasmid in Drug-Resistant Bacteria

**DOI:** 10.2174/0118715303352305250214071027

**Published:** 2025-03-20

**Authors:** Yanqing Tong, Jian Kang, Qian Wang

**Affiliations:** 1 Department of Nephrology, Affiliated Hospital to Changchun University of Chinese Medicine, Changchun 130021, China;; 2 Changchun University of Chinese Medicine, Changchun 130021, China;; 3 The Third Affiliated Hospital to Changchun University of Chinese Medicine, Changchun 130000, China

**Keywords:** *Staphylococcus aureus*, soft tissue infection, bacterial peritonitis, chinese medicine, antibacterial activity, resistant plasmid

## Abstract

**Background:**

*Staphylococcus aureus* is one of the most frequent human infections, which triggers various infectious diseases like soft tissue infection, lethal pneumonia, endocarditis, and bacteremia. The most common pathogen responsible for simple cystitis is *E.coli*; however, it also causes pneumonia, bacteremia, and abdominal infections, such as spontaneous bacterial peritonitis.

**Objective:**

Chinese medicines have been used effectively in the treatment of infectious disorders; thus, this study aimed to investigate the efficiency of Chinese medicine against *S. aureus*.

**Methods:**

An extract of traditional Chinese medicine was prepared using nine compounds: tongcao, talc, red peony root, fennel, guangui, lychee core, dry sunflower, dianthus, and purslane, to evaluate its antibacterial activity against *Staphylococcus aureus* RN450RF.

**Results:**

The minimum inhibitory concentration (MIC) of the Chinese medicine measured by the consecutive double dilution technique was 200g/L. The drug-resistant plasmid was transferred equally well under controlled laboratory conditions with a median conjugation frequency of 1.1x10^6^. The maximum activity of conjugated transfer of resistant drug plasmid of *E. coli* CP9 (R45) was observed at 2/1 MIC (100 g/L drug concentration), 32h time interval, with a bacterial concentration 10^8^ CFU/ml.

**Conclusion:**

These results suggest that the secondary inhibitory concentration (1/2 MIC) of the Chinese medicine solution can promote the combination and transfer of the resistance plasmid of Chinese medicine (R45) between different strains. The drug concentration, binding time, and initial bacterial concentration have different degrees of positive promotion effects on the conjugation and transfer of drug-resistant plasmids. Traditional Chinese medicine might be a potentially huge disease management and infection control resource.

## 1 INTRODUCTION

The infection of* staphylococcus aureus * ranges from common skin diseases like impetigo and furunculosis to serious deep-seated infections. *S. aureus* is regarded as the 1^st^ or 2^nd^ bacterial pathogen, which results in infections of the bloodstream [1]. It is also the leading cause of prosthetic implants, nosocomial pneumonia, and surgical wounds. Clinical isolates of methicillin-resistant *S. Aureus* (MRSA) have become the primary cause of severe infections globally [2].

Among the pathogenic bacteria and various life-threatening diseases, shock syndrome, endocarditis, and pneumonia are also associated with them. MRSA might account for more than half of all *S. aureus* strains in clinically infected individuals, resulting in enhanced mortality, healthcare burden, length of stay, and morbidity [3]. MRSA is not only resistant to β-lactam antibiotics but also to a wide range of antibiotic classes: macrolides, aminoglycosides, fluoroquinolones, and lincosamides. However, vancomycin is the most effective anti-MRSA agent till now, and it causes various adverse effects, such as ototoxicity, interstitial nephritis, red man syndrome, fever, hypotension, hypersensitivity reactions, neutropenia, phlebitis, and chills [4, 5]. The existence of resistant strains in MRSA and reduced vancomycin susceptibility has shown a significant clinical problem. Consequently, new treatment approaches to overcome MRSA infections and lessen MRSA selection pressure on present anti-MRSA drugs are unavoidably necessary [6, 7]. The most common infection responsible for uncomplicated cystitis is *E. coli*, but it also causes pneumonia, bacteremia, and abdominal ailments like spontaneous bacterial peritonitis. *E. coli* infection causes a significant burden on people and the healthcare system, so quick detection and therapy are essential [8, 9].

Consequently, developing novel and efficient antibiotics is required to avoid entering the “pre-antibiotic era” [10]. However, the decline in the discovery of new antibiotics has been observed in recent years due to diverse reasons, *i.e.*, regulatory hurdles, lack of significant government assistance, increased costs, and low investment returns [11, 12]. Chinese herbal medicines (CHMs), which have been utilized efficiently in several infectious illnesses for thousands of years in China, are considered a large resource for discovering new drugs [8, 9]. Some studies have reported that CHMs can successfully treat infectious diseases caused by drug-resistant bacteria. However, it is challenging to elucidate the exact information on the epidemiology of drug-resistant bacteria in healthcare and the overuse of antibiotics in China [13, 14].

The reports on the clinical efficacy of CHMs in the cure of infections caused by drug-resistant bacteria should be the elementary step in developing a new drug based on CHM for treating infections. However, in the current research, we report the *in vitro* antibacterial and synergistic effects of Chinese homemade medicine as conventional antibacterial substances against clinical isolates of *S. aureus*. We prepared extracts using nine medicinal plants, processed them, and then tested their antibacterial activities.

## 2 MATERIALS AND METHODS

### 2.1 Preparation of Plant Extract

For the preparation of Chinese medicine extract, all the raw materials *i.e.*, 4g of tongcao (Tetrapanax papyrifer), 20g of talc (hydrous magnesium silicate), 20g of red peony root (Paeonia lactiflora), 30g of fennel (Foeniculum vulgare), 15g of guangui, 30g of lychee core, 30g of dry sunflower, 40g of dianthus (Dianthus caryophyllus L), and 60g of purslane (Portulaca oleracea) were ground into a fine powder. The fine powder was intended for the proposed activity. Subsequently, 500ml of distilled water was added, and all the herbs were thoroughly soaked for 30 minutes and gently boiled for 30 min using a distillation process at 68-70 ^o^C. Carefully, the solution was poured into the beaker through the filter. The extract was centrifuged for 10 min at 12000 rppm, and the supernatant was taken. The concentrated solution was transferred into a sterile glass bottle and stored at -20^o^C for future use. All the extracts were conformed using chromatographic analysis.

### 2.2 Minimum Inhibitory Concentration Test

Bacteria were cultured as described previously [15]. Briefly, they were precultured in the nutrient broth (3 g of meat extract and 5 g of meat peptone in 1 L of deionized water at pH 7, which is the concentration of a broth suggested by the producer) for 3-4 h in a rotary shaker at 37 °C. By means of a spectrophotometer (Carry 50 UV-Vis Spectrophotometer, Agilent Technologies), the broth culture's turbidity was adjusted to 1 × 108 CFU/mL and diluted to a final inoculum having a concentration of around 1 × 105 CFU/mL. The spread-plate approach was used to test colony-forming units. Minimum inhibitory concentration (MIC) was calculated using the double dilution agar method suggested by the National Committee for Clinical Laboratory Standards (NCCLS). The agar medium was prepared with different drug concentrations at 200 g/L, 100 g/L, 50 g/L, 25 g/L, and 12.5 g/L. The tested strains were spotted with a multipoint incubation machine. Each Spot contained 10^4^ bacteria and was incubated at 35^o^C for 18-24h. Each batch of the experiment was compared with the standard strains. We obtained the MIC value of *E. coli* CP9 (carrying a drug-resistant plasmid R45); and *S. aureus* RN450RF [16], respectively.

### 2.3 Minimum Bactericidal Concentration (MBC) Test

After cultivating a purified culture of a particular microorganism overnight, it was attenuated in a growth-supporting broth (typically Mueller Hinton broth) to the desired concentration. Once the compound undergoing analysis was serially diluted, each dilution of the test product was inoculated with an equivalent volume of the designated microorganism. Following this, the microtiter plate or test tube was incubated for the specified time and temperature. Subsequent observations were conducted, during which turbidity indicated the presence of microorganisms, and the MIC, or minimum inhibitory concentration of bacterial growth, was not visually apparent. A dilution representing the MIC and at least two more concentrated dilutions of the test product were plated, and the survival CFU/ml was counted in order to determine the MBC. MBC should be at the lowest concentration capable of inhibiting the 99.9% activity of the tested microorganisms.

### 2.4 Resistant Plasmid Conjugation Transfer

Drug-resistant *E. coli* and *S. aureus* in LB media overnight were cultivated. The donor and recipient donor solution was adjusted to a 1:3 ratio by taking OD at 600nm. The culture was centrifuged at 4^o^C for 5 min at 5000rpm. The supernatant was discarded, and physiological saline was added to the original volume. About 5ml from each group was taken, and then an equal volume of serial dilutions of herbal concoction was added. In the blank control group, an equal volume of sterilized water was mixed well and incubated at 25^o^C. The experiment also established control group *E. coli* CP9 (R45) and *S. aureus* RN450RF control group to test whether the drug-resistant screened bacteria containing plates were all trans-conjugate, and all the *E. coli* CP9 and *S. aureus* RN450RF mutant were excluded. Each control group was added with the same drug concentration and the screening method was the same as the conjugate group.

### 2.5 Transconjugate Screening

The conjugate transfer was observed by diluting the binding solution 10 times to a concentration of 10^5^ CFU/ml. The transconjugants were screened using the agar plate method using 400 g/L drug with agar medium. The plates were placed at 37 ^o^C for 24h and observe whether the colonies were transconjugants.

### 2.6 Calculation of Conjugants at Different Drug Concentrations, Bacterial Concentrations, and Time

To ascertain the impact of the drug concentration on conjugation and transfer of resistance plasmid, 100 g/L and 200 g/L serial dilutions of herbal concoction were added to the culture solution, respectively, and incubated at 25°C for 8h. To ascertain the impact of conjugation time on conjugation and transfer of resistance plasmid, 100 g/L dilutions of herbal concoction were added to the culture solution and respectively incubated at 25°C for 8h, 32h, and 90h. To determine the impact of bacterial concentration on conjugation and transfer of resistance plasmid, the bacterial solution was diluted to 10^4^CFU/ml, 10^5^CFU/ml, 10^6^CFU/ml, 10^7^CFU/ml, and 10^8^CFU/ml, and then respectively incubated at 25°C for 8h for the calculation of conjugate number.

### 2.7 Statistical Analysis

Plant specimens were collected three times at each sampling site, and the mean as well as standard deviation were determined and tabulated. The findings of analysis were compared to those of the Standards Association of Zimbabwe.

## 3 RESULTS

### 3.1 Preparation of Chinese Medicines Extract

Chinese homemade medicine was prepared under ideal circumstances to achieve maximal recovery of Chinese medicine. Washing raw material with water eliminated the dust, soluble particles, and heavy pollutants, such as sand and dirt.

### 3.2 Minimum Inhibitory Concentration Test

MIC tests are usually used to determine the minimal dose of antimicrobials that will prevent microorganism growth after overnight incubation [17]. The determination results showed that the MIC of *E. coli* CP9 (MIC_1_) and *S. aureus* RN450RF (MIC_2_) were 200 g/L. The results of two MIC measurements showed that the growth of clinical test bacteria and quality control bacteria, as well as the antibacterial status of *Escherichia coli* and *Staphylococcus aureus*, are consistent. As shown in Fig. (**1**), there are no clinical strains that showed resistance to traditional Chinese medicine preparations.

The cold extract expressed the highest MIC, moreover, we also calculated the minimum bactericidal concentration (MBC). (Table **1**) enlists the MIC and MBC of *Escherichia coli* and *Staphylococcus aureus*.

### 3.3 Resistance Plasmid Conjugation Transfer

After incubation of drug resistance, the culture was completed, and the drug-resistant strains were screened using an MH agar medium with a concentration of 400 g/L Chinese medicine. The drug-resistant *E. coli* bacteria were isolated and preserved at -80^0^C. After conjugation, the number of conjugants was 1.1x10^6^.

### 3.4 Effect of Chinese Medicine on the Conjugation and Transfer of Drug Resistance Plasmid

The influence of various factors, such as drug concentration, conjugation time, and bacterial concentration, on the conjugative transfer of resistance plasmids was evaluated.

### 3.5 Effect of Drug Concentration on the Conjugation and Transfer of Drug Resistance Plasmid

A checkboard assay was combined with a drug concentration (100 g/L and 200 g/L) to determine the drug concentration on drug resistance plasmid transfer. The findings revealed that the most beneficial effect was found at a concentration of 100 g/L. The conjugal activity was decreased after increasing the drug concentration (Table **2**).

### 3.6 Effect of Time Intervals on the Conjugation and Transfer of Drug Resistance Plasmid

A checkerboard assay combined with a time was conducted to examine the effect for different time intervals for 8h, 32h, and 90h. The conjugative transfer of drug-resistant plasmid does not show a continuously increasing trend with the extension of the action time but first increases and then decreases. The minimum conjugate shift was observed at 8h, followed by 90h. In contrast, the maximum transfer was after 32h (Table **3**).

### 3.7 Effect of Bacterial Concentration on the Conjugation and Transfer of Drug Resistance Plasmid

The frequency of conjugative transfer of resistance plasmids between heterogeneous strains was evaluated when the bacterial concentration was from 10^4^cfu/mL to 10^8^cfu/mL. The data revealed that the maximum transfer was seen at 10^8^cfu/mL, followed by 10^7^cfu/mL. The minimum transfer of conjugative drug resistance plasmid was observed at 10^4^cfu/mL, followed by 10^7^cfu/Ml (Table **4**).

Moreover, we tested the toxicity and biocompatibility, and we found they do not have any serious adverse effects and are compatible with the host (data not shown).

## 4 DISCUSSION

The therapeutic effects of traditional Chinese herbal medicines against *S. aureus* and plasmid-mediated resistance of Chinese medicines have, to our knowledge, been the subject of few studies. It has been suggested that the combination of Chinese medicine and antibiotic therapy may yield more measurable outcomes against various diseases [18] than antibiotic therapy alone. Nonetheless, a variety of Chinese medicines are utilized, including tongcao, talc, red peony root, fennel, guangui, lychee core, dried sunflower, dianthus, and purslane. The liver is protected by various antibacterial, anti-inflammatory, and traditional Chinese medicine (TCM) properties of these botanicals [19].

To detect these two strains more sensitively, the difference in MIC was compared to ascertain the *in vitro* antibacterial activity of traditional Chinese medicine against *S. aureus*. The results of two MIC measurements showed that the minimum inhibitory concentrations of *E. coli* CP9 (MIC_1_) and *S. aureus* RN450RF (MIC_2_) were both 200 g/L. The growth of clinical tests and quality control bacteria and the antibacterial status of *E. coli* and *S. aureu*s were consistent. No clinical strains showed resistance to traditional Chinese medicine preparations. The MIC value reported in the present study against *S. aureus* was greater than the previously reported results [20, 21]. For instance, Jeyaseelan *et al*. reported that various extracts inhibited both *E. coli* and *S. aureus*. Cold and hot ethanol extracts inhibited *S. aureus* substantially more (*P*<0.05) than methanol extracts, with the hot ethanol extract having the lowest MIC and MBC values (5 mg/mL and 10 mg/mL, correspondingly) [22]. *E. coli* was substantially suppressed by hot ethanol and methanol extracts, with MIC and MBC values of 40 mg/mL and 80 mg/mL significantly. Oyewole *et al*. screened the antibacterial activities of a cold methanol extract of *R. communis* leaves against *S. aureus* and *E. coli* utilizing the broth dilution technique and found that the extract inhibited neither of the test bacteria [23]. The phytochemical analysis revealed that phlorotannins, tannins, terpenoids, flavonoids, and cardiac glucosides were present, but saponins were not. In the current study, however, methanol extracts inhibited growth using both the agar well diffusion technique and the broth dilution method. In addition, the phytochemical analysis revealed the existence of saponins but the absence of phlorotannins. The variance in outcomes might be attributable to differences in a variety of plants or geographical distribution [20]. Measuring the MICs facilitates detecting small changes in susceptibility that do not arise around the breakpoints of defined resistance. This method examined the significant differences in drug susceptibility. Routine MIC determination has numerous research and clinical benefits over disc diffusion testing and permits the pharmacodynamic/pharmacokinetic principles to be used when designing the dosing regimens on evidence-based [23]. After the screening of drug-resistant *E. coli* bacteria isolated at 400 g/L Chinese medicine after conjugation, the number of conjugants was 1.1x10^6^.

The transfer of R plasmids by conjugation mechanism among bacteria from various origins has been investigated in the present work. Conjugation is the well-studied and most significant mechanism for the gene transfer between bacteria [24]. To access the antimaterial activity of Chinese medicine against *S. aureus, *the effect of time, bacterial concentration, and drug concentration on the conjugal transfer of resistance plasmids was evaluated. The best conjugal transfer of resistance plasmid was observed at a concentration of 100 g/L, 10^8^cfu/mL, and 32h interval time. It is generally observed that the conjugal transfer of resistance plasmid appeared at higher bacterial concentrations and a long time. A previous study demonstrated that R plasmids carrying antimicrobial resistance can transfer between unrelated bacterial strains of human, animal, and fish origins in diverse natural environments, highlighting the environmental risk of spreading antimicrobial resistance, even without antibiotic presence, posing a global health threat [25]. We highlighted both the factors driving antimicrobial resistance, emphasizing environmental contamination and the impact of medicinal compounds on plasmid-mediated resistance transfer.

The study did not include standard antibiotic controls; however, future investigations will incorporate antibiotics, such as penicillin or ciprofloxacin, for comparative efficacy analysis. *E. coli* CP9 (MIC_1_) was selected due to its clinical relevance and plasmid-mediated resistance, while *S. aureus* RN450RF was chosen for its significance as a human pathogen causing diverse infections. These strains provide a robust model for evaluating antibacterial activity and plasmid transfer dynamics.

Based on the reported results, significant study perspectives are needed to better evaluate the safety and effectiveness of applied Chinese medicine in identifying and protecting *S. aureus* infection. It is essential to have a validated and reliable prescription, and future trials are required.

## 5 LIMITATIONS

The empirical results reported here should be considered in light of some limitations. Given that this project utilizes the effective components with in vitro antibacterial activity from a "traditional Chinese medicine extract" (prepared using nine herbs: Tongcao, Talcum, Red Peony Root, Fennel, Guanggui, Lychee Seed, Dried Sunflower, Dendrobium, and Purslane), we have cultured and screened *E. coli* CP9 and *S. aureus* RN450RF strains that are resistant to traditional Chinese medicines. Consequently, the resistant strains cultivated may only exhibit resistance to the herbs in the "traditional Chinese medicine extract". Therefore, the research findings of this project solely represent the study on *E. coli* CP9 and *S. aureus* RN450RF strains resistant to the active ingredients of the "traditional Chinese medicine extract".

## CONCLUSION

In conclusion, this investigation indicates that cold and hot extracts are viable sources for *E. coli* and *S. aureus*. Even at lower concentrations, the cold and hot extracts of ethanol are more effective against *S. aureus*. Additional research is required to determine the exact bioactive constituents, their mode of action, and their safety *in vivo*. The existence of the secondary inhibitory concentration (1/2 MIC) of the Chinese medicine solution can promote the combination and transfer of the resistance plasmid of Chinese medicine (R45) between different strains. The drug concentration, binding time, and initial bacterial concentration have different degrees of positive promotion effects on the conjugation and transfer of drug-resistant plasmids. Traditional Chinese medicine might be a potentially huge disease management and infection control resource.

There is still more work to be done in the field of EO antimicrobial usage, namely in terms of procedure standardization, threshold definition, and guidance, which are essential measures for obtaining reliable data and building robust sensitivity/resistance profiles of Chinese medicine compounds to determine the antibacterial activity against *S. aureus* RN450RF and beyond.

## Figures and Tables

**Fig. (1):**
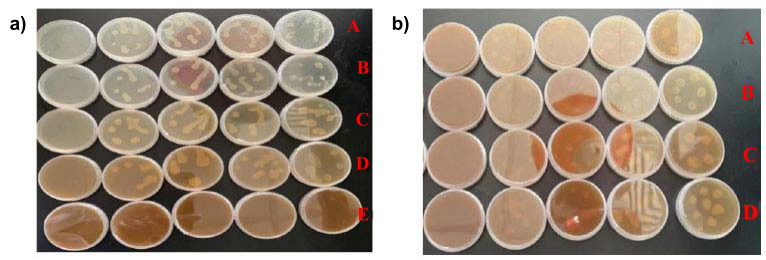
**a)** Showed the MIC test measurement **A)** quality measurement of *Staphylococcus aureus* **B)** quality control of *Escherichia coli* **C)** clinical strain of *Staphylococcus aureus* **D)** clinical strain of *Escherichia coli* **E)** control **b)** showed the MIC test measurement **A)** clinical strain of *Staphylococcus aureus* **B)** *Staphylococcus aureus * quality control **C)** clinical strain of *Escherichia coli* **D)** quality control of *Escherichia coli * (concentration of drug solution from left to right are 200 g/L, 100 g/L, 50 g/L, 25 g/L and 12.5 g/L in parallel 2 boards).

**Table 1:** MBC and MIC of test extracts (g/L) of *Staphylococcus aureus* and *Escherichia coli*.

-	***E. coli***		***S. aureus***	
-	**MIC**	**MBC**	**MIC**	**MBC**
Cold Extract	100	200	100	200
Hot Extract	50	100	50	100

**Table 2:** Influence of different drug concentrations on the conjugation and transfer of drug resistance plasmid.

**Drug Concentration**	**100 g/L**	**200 g/L**
**Conjugate number 1.1x10^6^**	2.40 ± 0.26	1.19 ± 0.15

**Table 3:** Influence of time interval on the conjugation and transfer of drug resistance plasmid.

**Drug Concentration**	**8h**	**32h**	**90h**
**Conjugate number 1.1x10^6^**	1.18 ± 0.13	1.77 ± 0.09	1.51 ± 0.12

**Table 4:** Influence of bacterial concentration on the conjugation and transfer of drug resistance plasmid.

**Drug Concentration**	**10^4^cfu/mL**	**10^5^cfu/mL**	**10^6^cfu/mL**	**10^7^cfu/mL**	**10^8^cfu/mL**
**Conjugate number 1.1x10^6^**	14.33 ± 4.04	104.67 ± 9.45	1252 ± 196.52	14761.33 ± 1705.53	118502 ± 14707.23

## Data Availability

The data and supportive information are available within the article.

## LIST OF ABBREVIATIONS

MIC: Minimum Inhibitory Concentration
NCCLS: National Committee for Clinical Laboratory Standard
MRSA: Methicillin-Resistant *S. Aureus*
CHMs: Chinese Herbal Medicines

## References

[1] Usery J.B., Vo N.H., Finch C.K., Cleveland K.O., Gelfand M.S., Self T.H. (2015). Evaluation of the treatment of methicillin-resistant *Staphylococcus aureus* bacteremia.. Am. J. Med. Sci..

[2] Zingg W., Holmes A., Dettenkofer M., Goetting T., Secci F., Clack L., Allegranzi B., Magiorakos A.P., Pittet D. (2015). Hospital organisation, management, and structure for prevention of health-care-associated infection: A systematic review and expert consensus.. Lancet Infect. Dis..

[3] Leimbach A., Hacker J., Dobrindt U. (2013). *E. coli* as an all-rounder: The thin line between commensalism and pathogenicity.. Curr. Top. Microbiol. Immunol..

[4] Köhler C.D., Dobrindt U. (2011). What defines extraintestinal pathogenic *Escherichia coli*?. Int. J. Med. Microbiol..

[5] Bruniera F.R., Ferreira F.M., Saviolli L.R., Bacci M.R., Feder D., da Luz Gonçalves Pedreira M., Sorgini Peterlini M.A., Azzalis L.A., Campos Junqueira V.B., Fonseca F.L. (2015). The use of vancomycin with its therapeutic and adverse effects: A review.. Eur. Rev. Med. Pharm..

[6] Hiramatsu K., Hanaki H., Ino T., Yabuta K., Oguri T., Tenover F.C. (1997). Methicillin-resistant *Staphylococcus aureus* clinical strain with reduced vancomycin susceptibility.. J. Antimicrob. Chemother..

[7] Xiao-Meng W., Xiao-Bo L.I., Ying P. (2017). Impact of Qi-invigorating traditional Chinese medicines on intestinal flora: A basis for rational choice of prebiotics.. Chin. J. Nat. Med..

[8] Guo Y., Song G., Sun M., Wang J., Wang Y. (2020). Prevalence and therapies of antibiotic-resistance in *Staphylococcus aureus*.. Front. Cell. Infect. Microbiol..

[9] Jiang M., Zha Q., Zhang C., Lu C., Yan X., Zhu W., Liu W., Tu S., Hou L., Wang C., Zhang W., Liang Q., Fan B., Yu J., Zhang W., Liu X., Yang J., He X., Li L., Niu X., Liu Y., Guo H., He B., Zhang G., Bian Z., Lu A. (2015). Predicting and verifying outcome of Tripterygium wilfordii Hook F. based therapy in rheumatoid arthritis: From open to double-blinded randomized trial.. Sci. Rep..

[10] Kuok C.F., Hoi S.O., Hoi C.F., Chan C.H., Fong I.H., Ngok C.K., Meng L.R., Fong P. (2017). Synergistic antibacterial effects of herbal extracts and antibiotics on methicillin-resistant *Staphylococcus aureus* : A computational and experimental study.. Exp. Biol. Med. (Maywood).

[11] Robertson F., Jagers S., Rönnerstrand B. (2018). Managing sustainable use of antibiotics: The role of trust.. Sustainability (Basel).

[12] Neuman H., Forsythe P., Uzan A., Avni O., Koren O. (2018). Antibiotics in early life: Dysbiosis and the damage done.. FEMS Microbiol. Rev..

[13] Cao Y., Wu K., Mehta R., Drew D.A., Song M., Lochhead P., Nguyen L.H., Izard J., Fuchs C.S., Garrett W.S., Huttenhower C., Ogino S., Giovannucci E.L., Chan A.T. (2018). Long-term use of antibiotics and risk of colorectal adenoma.. Gut.

[14] Kumar M., Jaiswal S., Sodhi K.K., Shree P., Singh D.K., Agrawal P.K., Shukla P. (2019). Antibiotics bioremediation: Perspectives on its ecotoxicity and resistance.. Environ. Int..

[15] Štumpf S., Hostnik G., Primožič M., Leitgeb M., Salminen J.P., Bren U. (2020). The effect of growth medium strength on minimum inhibitory concentrations of tannins and tannin extracts against. *E. coli*.. Molecules.

[16] Tong Y.Q., Xin B., Zhu L. (2014). Transfer of herb-resistance plasmid from *Escherichia coli* to *Staphylococcus aureus* residing in the human urinary tract.. Jundishapur J. Microbiol..

[17] Annis D.H., Craig B.A. (2005). Statistical properties and inference of the antimicrobial MIC test.. Stat. Med..

[18] Yang W., Liu J., Blažeković B., Sun Y., Ma S., Ren C., Vladimir-Knežević S. (2018). *In vitro* antibacterial effects of Tanreqing injection combined with vancomycin or linezolid against methicillin-resistant *Staphylococcus aureus*.. BMC Complement. Altern. Med..

[19] Yokota J. (2017). Application of natural ingredients to preventive medicine.. Yakugaku Zasshi.

[20] Dong D., Ni Q., Wang C., Zhang L., Li Z., Jiang C., EnqiangMao, Peng Y. (2018). Effects of intestinal colonization by Clostridium difficile and *Staphylococcus aureus* on microbiota diversity in healthy individuals in China.. BMC Infect. Dis..

[21] Ma Y., Chen M., Guo Y., Liu J., Chen W., Guan M., Wang Y., Zhao X., Wang X., Li H., Meng L., Wen Y., Wang Y. (2019). Prevention and treatment of infectious diseases by traditional Chinese medicine: A commentary.. Acta. Pathol. Microbiol. Scand. Suppl..

[22] Jeyaseelan E.C., Jashothan P.T.J. (2012). *In vitro* control of *Staphylococcus aureus* (NCTC 6571) and *Escherichia coli* (ATCC 25922) by *Ricinus communis* L.. Asian Pac. J. Trop. Biomed..

[23] Wu M., Li Y., Guo D., Kui G., Li B., Deng Y., Li F. (2018). Microbial diversity of chronic wound and successful management of traditional Chinese medicine.. Evid. Based Complement. Alternat. Med..

[24] Wan Z., Varshavsky J., Teegala S., McLawrence J., Goddard N.L. (2011). Measuring the rate of conjugal plasmid transfer in a bacterial population using quantitative PCR.. Biophys. J..

[25] Kruse H., Sørum H. (1994). Transfer of multiple drug resistance plasmids between bacteria of diverse origins in natural microenvironments.. Appl. Environ. Microbiol..

